# An Unusual Variation of the Accessory Nerve

**DOI:** 10.7759/cureus.2774

**Published:** 2018-06-09

**Authors:** Yusuf Alimi, Joe Iwanaga, Marios Loukas, Rod J Oskouian, R. Shane Tubbs

**Affiliations:** 1 Anatomy, St. George's University School of Medicine, Grenada, West Indies, Seattle, USA; 2 Seattle Science Foundation, Seattle, USA; 3 Department of Anatomical Sciences, St. George's University, School of Medicine, Przemyśl, GRD; 4 Neurosurgery, Swedish Neuroscience Institute, Seattle, USA; 5 Neurosurgery, Seattle Science Foundation, Seattle, USA

**Keywords:** accessory nerve, variations, internal jugular vein, sternocleidomastoid muscle

## Abstract

The accessory nerve is an important nerve in the head and neck regions. Some variants of this nerve’s anatomy have been reported. Herein, we present an unusual report and review the extant medical literature regarding other more commonly found derailments of this nerve’s anatomy.

## Introduction

The accessory nerve is the eleventh of the 12 cranial nerves. It possesses both cranial and spinal root components with the latter originating from the lateral gray matter of the C1 to C5 parts of the spinal cord [1-5]. The cervical spine contribution enters the skull through the foramen magnum and coalesces with the cranial roots to form a trunk which exits the skull through the jugular foramen [4-5]. After exiting the skull, the accessory nerve descends into the neck and branches into the sternocleidomastoid (SCM) and trapezius muscles [1-5]. 

## Case presentation

**Figure 1:**
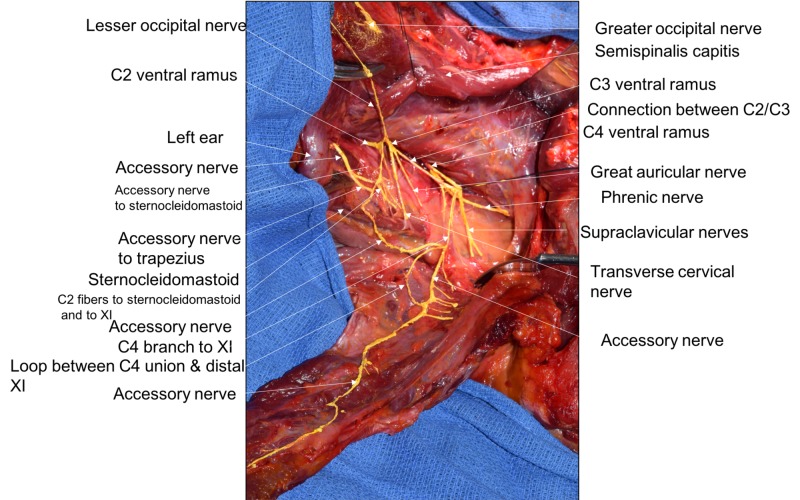
Posterior dissection of the left lateral neck Trapezius is reflected from its midline and scapular attachments and moved superolaterally to view its deep surface.

**Figure 2:**
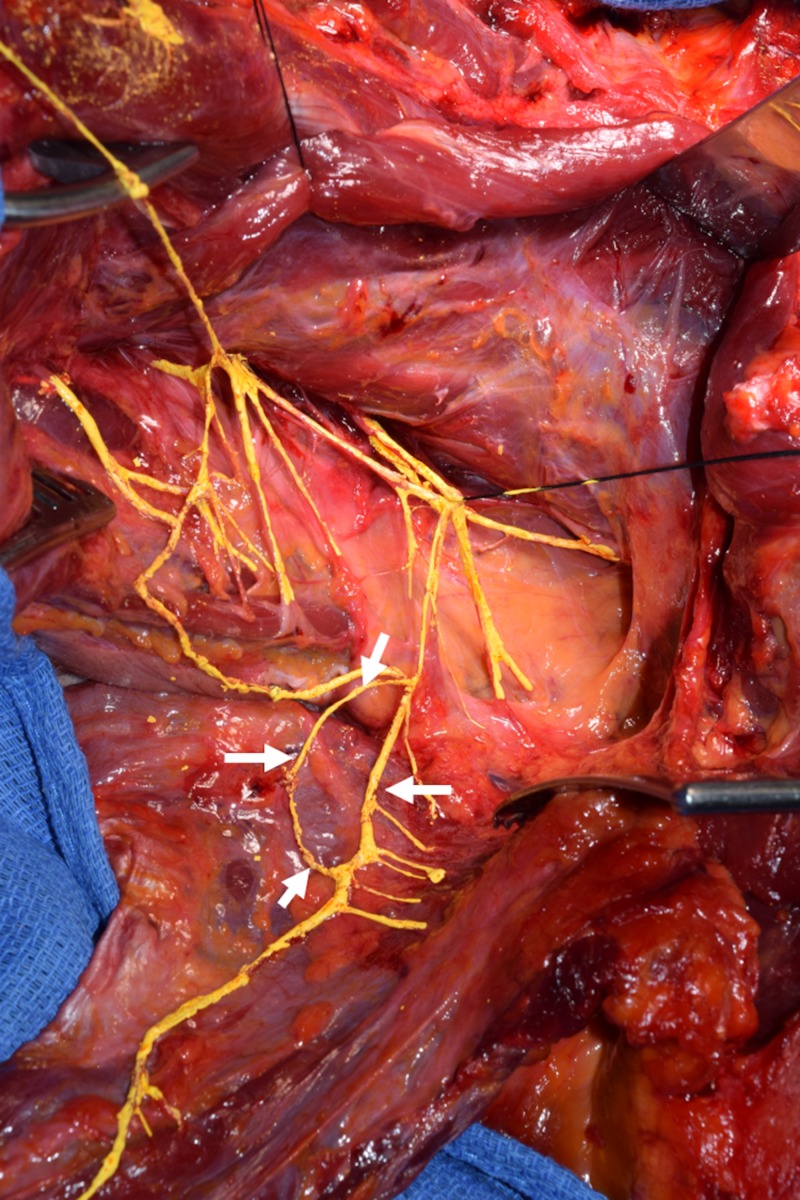
Repeat of Figure 1 emphasizing the neural loop (arrows) formed on the deep surface of the trapezius muscle and made up of one-half of distal fibers of the C4 ventral ramus and one-half from the distal accessory nerve to the trapezius

During the routine dissection of the head and neck region in a fresh frozen female cadaver who had died of heart failure at the age of 87 years, an unusual accessory nerve was observed on the left side (Figure 1). With a reflection of the trapezius muscle, an unusual loop was seen formed partially by the accessory nerve to the trapezius and partially by a continued branch of the C4 ventral ramus branch to the trapezius muscle (Figures 1, 2). No branches were seen emanating from the C4 ventral ramus of the loop; however, branches to the trapezius from the adjacent accessory nerve part of the loop were seen and were six in number (Figure 2). No similar nerve loop was identified on the contralateral side and no other obvious gross anatomical variants were seen in the areas dissected.

## Discussion

We identified an unusual nerve loop formed deep to the trapezius and made up of fibers half derived from a distal continuation of the ventral ramus of C4 and half from the accessory nerve deep to the trapezius. Variations in the path of descent taken by the accessory nerve and its branches after exiting the skull have been described in the literature. Spinal contribution to the accessory nerve originates near the C1 to C5 anterior and posterior rootlets [2, 4-6]. Four variations of the accessory nerve in terms of its intradural communication with the C1 nerve rootlets have been observed [6]. The first variant (type I) has no posterior root of C1 and the accessory nerve sometimes connects to the anterior rootlets. In the second variant (type II), there is no connection between the accessory nerve and the C1 posterior rootlets. The third variant (type III) involves a connection between the C1 posterior rootlets and the accessory nerve at their point of intersection or via an anastomotic division of the C1 posterior rootlets. The fourth type (type IV) involves the interaction between the accessory nerve and the C1 posterior root, which is attached to the spinal cord [6]. Variations in the number of branches received by the accessory nerve from the cervical plexus have been reported  [2, 5].

There also seems to be a significant degree of variation among individuals when comparing the relationship of the accessory nerve to the internal jugular vein (IJV). Saman et al. reported on the relationship between the accessory nerve and the IJV in the anterior neck [1]. They performed neck dissections of 61 cadaveric specimens and followed the path of the accessory nerve from the base of the skull to the anterior cervical triangle. It was observed that the accessory nerve starts anteromedial to the IJV within the jugular foramen in 87% of the specimen. The nerve then exits the jugular foramen on the lateral position to the IJV in 67% of necks dissected. Finally, in the anterior triangle of the neck, the accessory nerve starts at first medially to the IJV and later during its course, the majority of the specimens (80%) had the accessory nerve cross anteriorly to the IJV [1]. A similar study was performed by Tubbs et al. with results showing that a majority of the cadaveric specimens had an accessory nerve that crossed the IJV anteriorly [5]. Other cases of the accessory nerve piercing the IJV have been reported [1, 3]. Saman et al. measured the distance that the accessory nerve traveled from its exit at the jugular foramen to it crossing the IJV [1].

Variations in the number of branches originating from the accessory nerve terminating in the trapezius are common as well as differences in the course taken by the nerve in the posterior cervical triangle [2-3, 5]. Other variations that have been reported in the literature include duplication of the accessory nerve intracranially, the connection of the accessory and facial nerves with both providing innervations to the SCM, and contributing to the inferior root of the ansa cervicalis [5, 7]. Anastomotic connections between the cervical plexus and branches of the accessory nerve, known as Maubrac’s ansa, have also been reported to provide innervation to the SCM and trapezius [8]. However, this was not represented in our case.

Such anatomical variations are important for physicians to understand when evaluating patients with unusual physical examination findings [9]. Moreover, since there is no guarantee that a patient has a “normal” accessory nerve anatomy, it is important that surgeons are aware of the types of variations that might be encountered when working in the vicinity of this nerve so as to prevent unwanted complications [10-12].

## Conclusions

The accessory nerve is undoubtedly one of the most important nerves in the head and neck region. Its tortuous and variable anatomy can provide challenges when operating along its path and, if severed, can lead to complications, such as shoulder syndrome with symptoms consisting of shoulder pain, SCM, and trapezius muscle atrophy, winging of the scapula, as well as drooping of the affected shoulder, all of which leads to a decrease in the quality of life of the patient. Iatrogenic injury is one of the major causes of accessory nerve damage, and therefore, the need for a greater understanding of its existing variations, as well as awareness of unusual variants, such as in our case, cannot be understated.
